# Supplementary material for: Timing of antibiotic administration determines the spread of plasmid-encoded antibiotic resistance during microbial range expansion
Source: Nat Commun. 2023 Jun 14;14:3530. doi: 10.1038/s41467-023-39354-z (PMC10267205; doi:10.1038/s41467-023-39354-z)
Supplement: Supplementary file 1 — Supplementary Information [file 41467_2023_39354_MOESM1_ESM.pdf]

Supplementary Information

Timing of antibiotic administration determines the spread of plasmid-encoded antibiotic resistance during microbial range expansion

Authors
Yinyin Ma[1,2*], Josep Ramoneda[1,3] & David R. Johnson[1,4*]

Affiliations
[1]Department of Environmental Microbiology, Swiss Federal Institute of Aquatic Science and Technology (Eawag), 8600 Dübendorf, Switzerland; [2]Department of Environmental Systems Science, Swiss Federal Institute of Technology (ETH), 8092 Zürich, Switzerland; [3]Cooperative Institute for Research in Environmental Sciences (CIRES), University of Colorado, Boulder, CO 80309, USA; [4]Institute of Ecology and Evolution, University of Bern, 3012 Bern, Switzerland.

Correspondence:
Yinyin Ma; Email: yinyin.ma@eawag.ch
David R. Johnson; Email: david.johnson@eawag.ch

This file includes:
Supplementary Tables 1 and 2
Supplementary Figures 1-4

Supplementary Table 1: Measurements from the collision assays for the pAR145 donor and potential recipient strains.

Replicate	s	R_b	l
1	0.834	207.552	268.231
2	0.84	230.751	298.679
3	0.827	230.756	295.293
4	0.83	230.742	295.799

s is the selective advantage, or in our case the cost that pAR145 confers. R_b is the radius of the circle generated by the arc at the collision boundary. l is the distance between the two colonies.

Supplementary Table 2: Strains and plasmids used in this study.

Strain or plasmid	Relevant characteristics	Supplementary Reference
P. stutzeri A1601ech	A1501 with Δ*comA,* Δ*narG* and mini-Tn7T-LAC-Gm-*echerry*; Gm^R, $echerry^+$	1, 2
P. stutzeri A1601gfp	A1501 with Δ*comA*, Δ*nirS* and mini-Tn7T-LAC-Gm-*egfp*; Gm^R, $egfp^+$	1, 2
plasmid pAR145ecfp	pSU2007 *aph::cat*-PA1/04/03-*cfp*/-T0	3, 4

a b

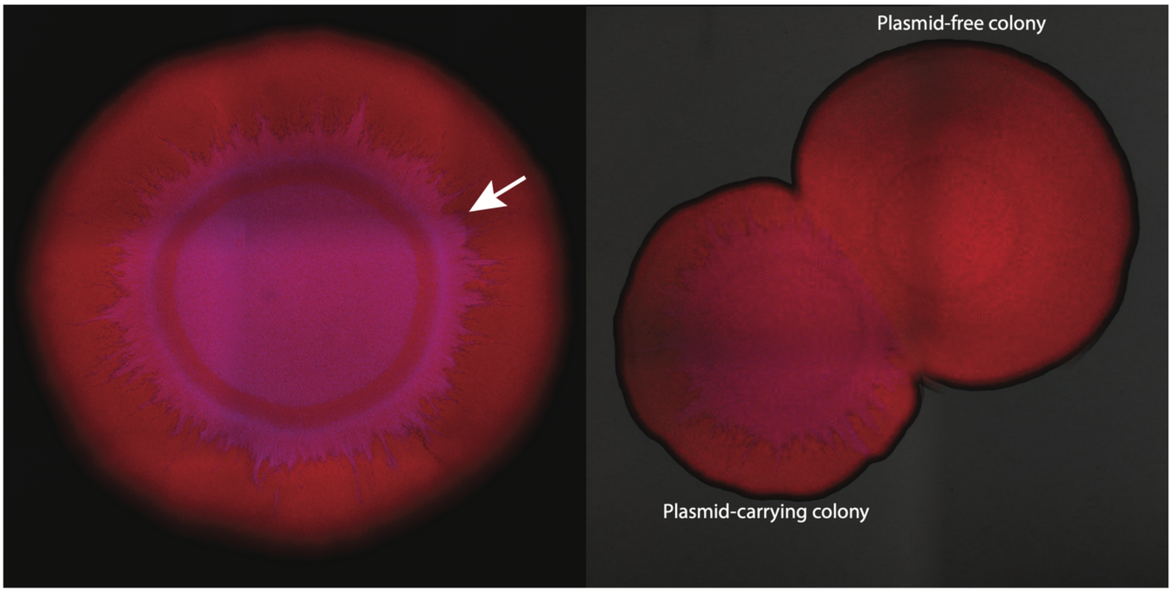

Supplementary Fig. 1: Loss of pAR145 in the absence of chloramphenicol pressure during range expansion. a Range expansion of a single pAR145-carrying strain that initially expresses red fluorescent protein from its chromosome and blue fluorescence from pAR145 (appears magenta). The white arrow indicates a pAR145 loss event indicated by a change in color from magenta to red. **b** We performed the colony collision experiment between *P. stutzeri* A1601ech with pAR145 (magenta) and *P. stutzeri* A1601ech (red). We inoculated the two strains at the same time at a distance of approximately 3 mm from each other. We took images after 96 hours of range expansion at room temperature. The smaller size of the pAR145-carrying colony indicates a slower expansion rate, which demonstrates that carrying pAR145 is costly in the absence of chloramphenicol pressure.

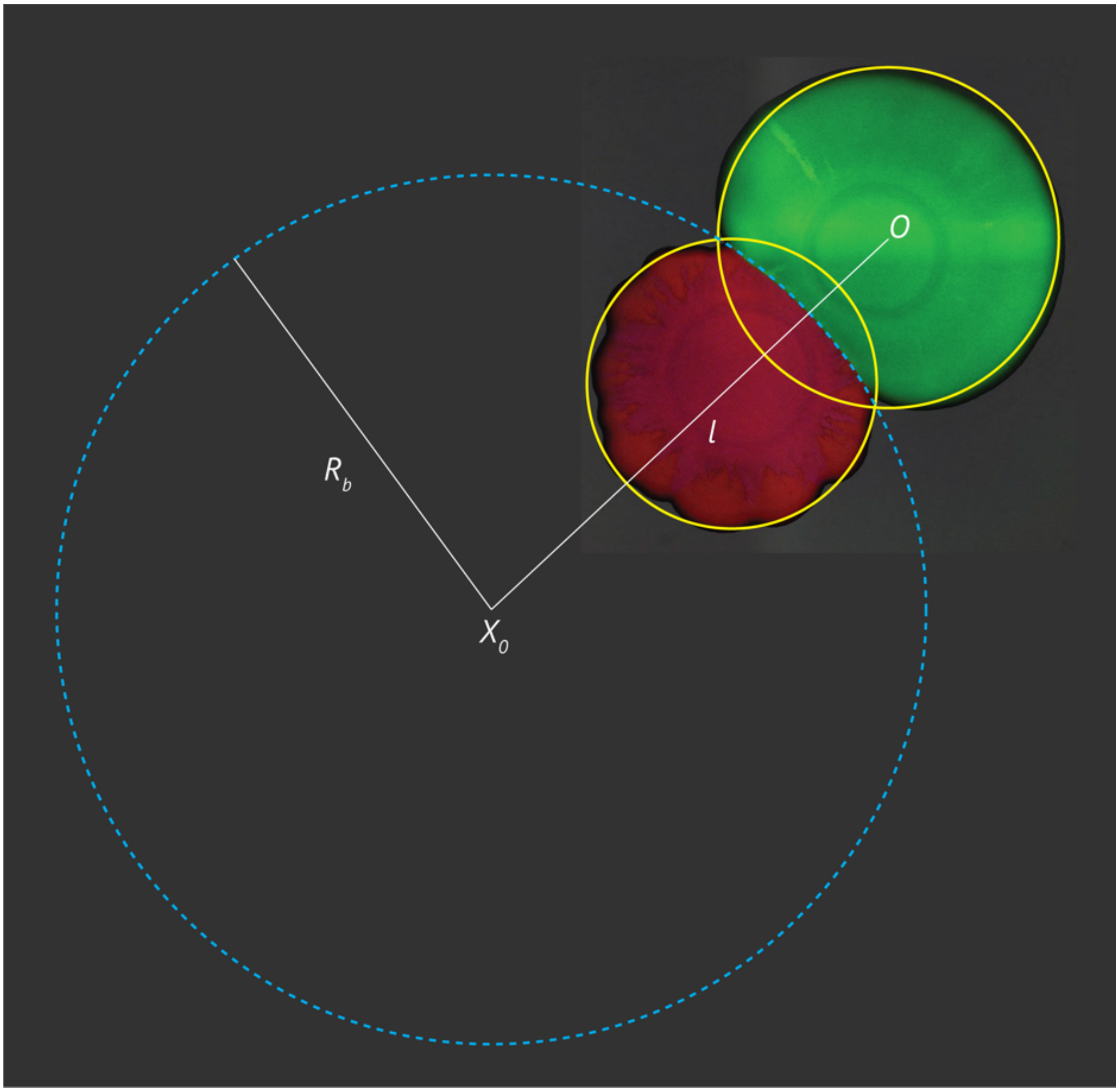

Supplementary Fig. 2: Collision assay of *P. stutzeri* A1601ech containing pAR145 and *P. stutzeri* A1601gfp. The geometry of the colonies and the collision plane can be used to quantify the relative growth rates of the two strains as described by Korolev *et al*.[71] We drew the larger circle based on the arc generated between the two colonies. X_0 and O are the centroids of the derived circles. l is the distance between X_0 and O. R_b is the radius of the derived circle.

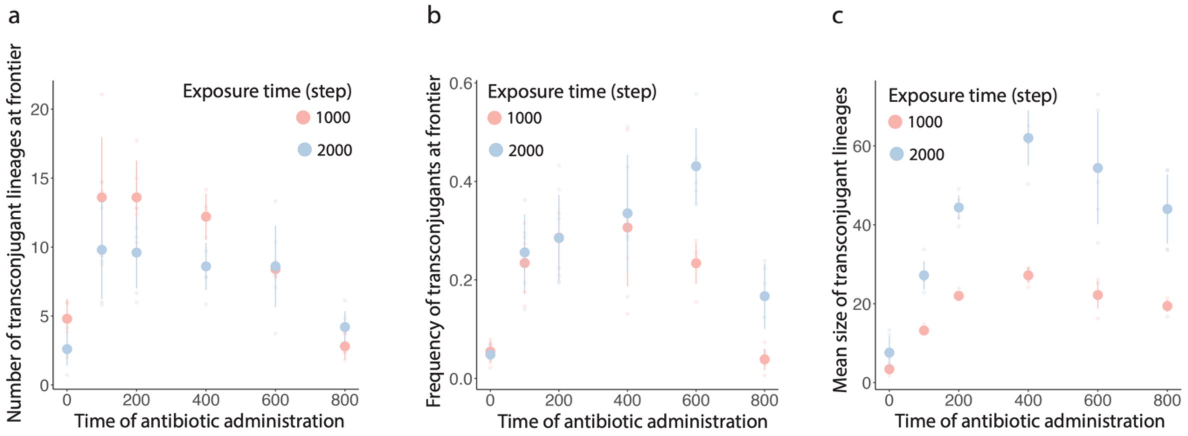

Supplementary Fig. 3: Simulation results for prolonged range expansion times (2000 time steps) and comparison to the standard range expansion time (1000 time steps). Light red and light blue data points are for exposure times to “antibiotics” of 1000 time steps and 2000 time steps, respectively. Effect of antibiotic administration time on **a** the number of transconjugant lineages at the expansion frontier, **b** the frequency of transconjugants at the expansion frontier, and **c** the mean size of transconjugant lineages. For **a**-**c**, all quantities are measurements at the end of the simulation times, data points are the means for each treatment, and error bars are 95% confidence intervals (n = 5).

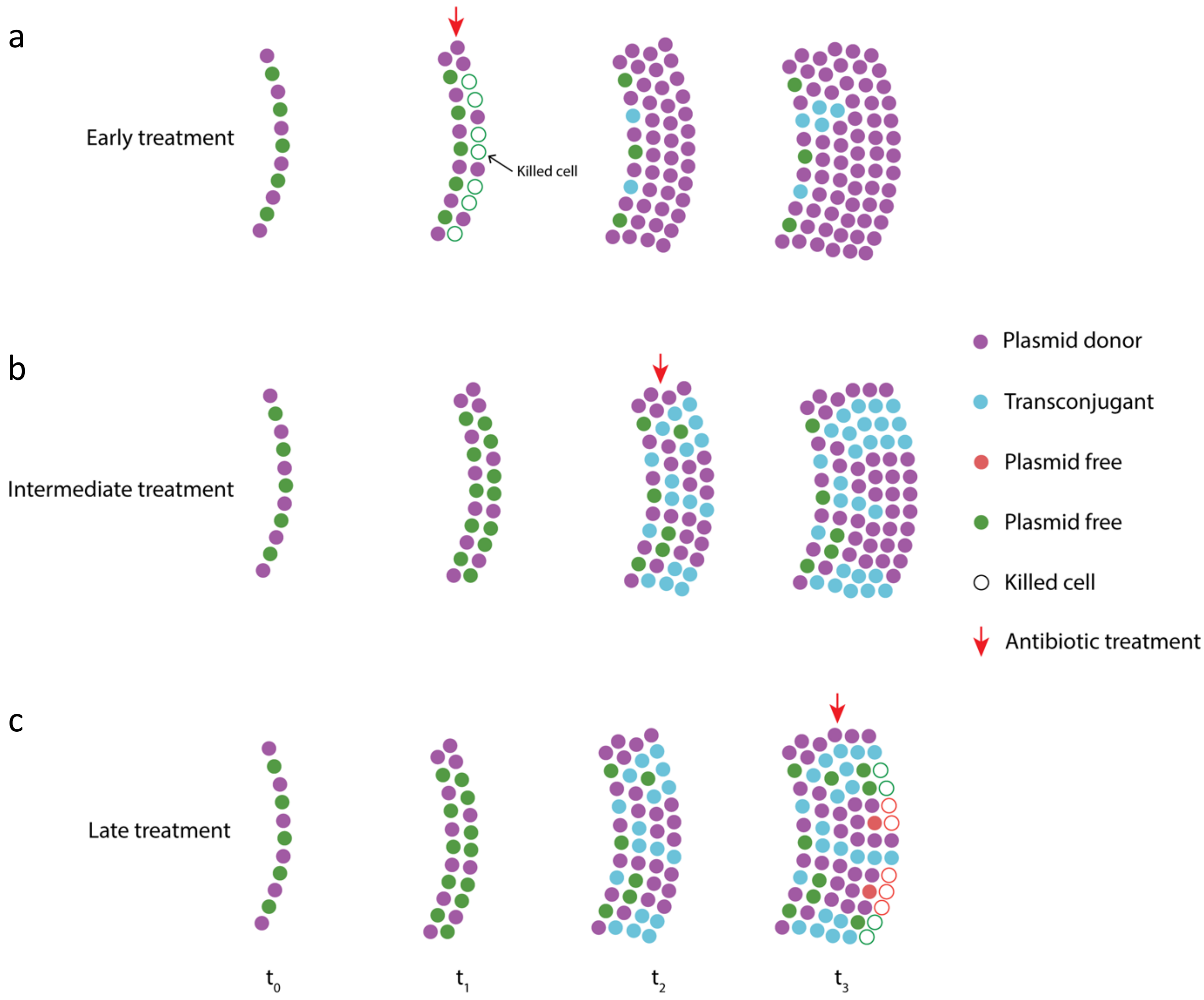

Supplementary Fig. 4: Schematic of transconjugant proliferation dynamics for different antibiotic administration times. Each colored circle represents one cell. Magenta cells are plasmid donor cells and green cells are potential recipient cells. When green cells receive the plasmid, they turn cyan. Cells expand from left to right from t_0 to t_3. Plasmid transfer does not occur before t_2 and plasmid loss does not occur before t_3. Empty circles indicate cells that are killed by antibiotic treatment. Red arrows indicate the time antibiotic administration. **a** When the antibiotic is applied at an early phase (t_1) before transconjugant cells are formed, plasmid donors will dominate the expansion frontier (magenta cells). **b** When the antibiotic is applied at intermediate phase (t_2) after plasmid transfer has begun, new transconjugant cells (cyan) can successfully proliferate along with the plasmid donors. Both plasmid donor and transconjugant cells will occupy the expansion frontier. **c** When the antibiotic is applied at a later phase (t_3) after plasmid loss has begun, both new transconjugant and plasmid donor cells will have reverted back to the plasmid-free state. These cells cannot grow after antibiotic administration.

Supplementary References

1. Lilja, E. E. & Johnson, D. R. Metabolite toxicity determines the pace of molecular evolution within microbial populations. *BMC Evol. Biol.* **17**, 52 (2017).

2. Lilja, E. E. & Johnson, D. R. Segregating metabolic processes into different microbial cells accelerates the consumption of inhibitory substrates. *ISME J.* **10**, 1568-1578 (2016).

3. Reisner, A., Molin, S. & Zechner, E. L. Recombinogenic engineering of conjugative plasmids with fluorescent marker cassettes. *FEMS Microbiol. Ecol.* **42**, 251-259 (2002).

4. Reisner, A., Wolinski, H. & Zechner, E. L. In situ monitoring of IncF plasmid transfer on semi-solid agar surfaces reveals a limited invasion of plasmids in recipient colonies. *Plasmid* **67**, 155-161 (2012).
